# Deletion of Munc18-1 in 5-HT Neurons Results in Rapid Degeneration of the 5-HT System and Early Postnatal Lethality

**DOI:** 10.1371/journal.pone.0028137

**Published:** 2011-11-29

**Authors:** Jacobus J. Dudok, Alexander J. A. Groffen, Ruud F. T. Toonen, Matthijs Verhage

**Affiliations:** Department of Functional Genomics, Center for Neurogenomics and Cognitive Research (CNCR), Neuroscience Campus Amsterdam, VU University, Amsterdam, The Netherlands; INSERM U901, France

## Abstract

The serotonin (5-HT) system densely innervates many brain areas and is important for proper brain development. To specifically ablate the 5-HT system we generated mutant mice carrying a floxed Munc18-1 gene and Cre recombinase driven by the 5-HT-specific serotonin reuptake transporter (SERT) promoter. The majority of mutant mice died within a few days after birth. Immunohistochemical analysis of brains of these mice showed that initially 5-HT neurons are formed and the cortex is innervated with 5-HT projections. From embryonic day 16 onwards, however, 5-HT neurons started to degenerate and at postnatal day 2 hardly any 5-HT projections were present in the cortex. The 5-HT system of mice heterozygous for the floxed Munc18-1 allele was indistinguishable from control mice. These data show that deletion of Munc18-1 in 5-HT neurons results in rapid degeneration of the 5-HT system and suggests that the 5-HT system is important for postnatal survival.

## Introduction

The 5-HT system consists of clusters of cell bodies in the midbrain raphe nuclei, with the largest clusters in the median raphe nucleus and the dorsal raphe nucleus (DRN). Several brain areas receive dense 5-HT innervation and 5-HT is released both synaptically and as volume transmission [1], [2]. Due to this and to the several 5-HT receptor subtypes which are present in the brain, 5-HT has many roles and influences many processes in the brain [3].

Neurogenesis of 5-HT neurons in the mouse brain occurs in the ventral rhombencephalon around embryonic day (E) 10 [4]. One day later, 5-HT neurons begin to synthesize and secrete 5-HT and start growing out axons. Around birth, target areas such as the forebrain and the hippocampus are densely innervated with 5-HT projections. Only after birth, the maturation of the 5-HT network is completed.

Several studies have addressed the role of 5-HT on the development of the 5-HT system and brain development. In a conditional Lmx1b knockout (KO) mouse, almost all 5-HT neurons fail to survive, resulting in a significant decrease in brain tissue 5-HT levels [5]. However, these mice do not show an overt phenotype and survive to adulthood [5]. In contrast, it was shown that maternal 5-HT is required for embryonic development [6]. Furthermore, in tryptophan hydroxylase 2 (Tph2) KO mice 5-HT neurons are completely devoid of 5-HT, but the morphology and neurite distribution of the 5-HT system is not affected and these mice do show only a subtle behavioural phenotype [7], [8], [9]. Neonatal depletion of 5-HT by the neurotoxin 5,7-Dihydroxytryptamine results in rather subtle changes in behavioural response and brain development [10], [11].

In this study we silenced the 5-HT system by conditional deletion of Munc18-1 in 5-HT neurons. Munc18-1 is a presynaptic protein which is essential for vesicle release and neurons that lack Munc18-1 have a complete absence of neurotransmitter secretion [12]. Via interactions with Syntaxin1A and the SNARE complex, Munc18-1 is involved in vesicle docking and fusion [13]. Munc18-1 knockout mice are born paralyzed and die immediately after birth [12]. In these mice, initially synapses are formed and the assembly of the brain is normal. However, in later stages of brain development there is massive neuronal cell death and brain degeneration [12]. Since Munc18-1 knockout mice die immediately after birth, we have generated Munc18-1^lox/lox^ mutant mice in order to conditionally delete Munc18-1. Crossing these mice with a L7-Cre line, with Cre expressed in Purkinje neurons in the cerebellum, resulted in mice which developed severe ataxia, suggesting a cerebellar phenotype [14].

In SERT-Cre^cre/wt^ Munc18-1^lox/lox^ mice 5-HT neurons were initially generated and 5-HT projections innervated the midbrain and cortex, later followed by degeneration and loss of 5-HT projections in the cortex. The majority of these mice died within a few days after birth. These data suggest that the 5-HT system contributes importantly to postnatal brain development.

## Results

### Deletion of Munc18-1 in SERT expressing neurons results in postnatal lethality

**Figure 1:**
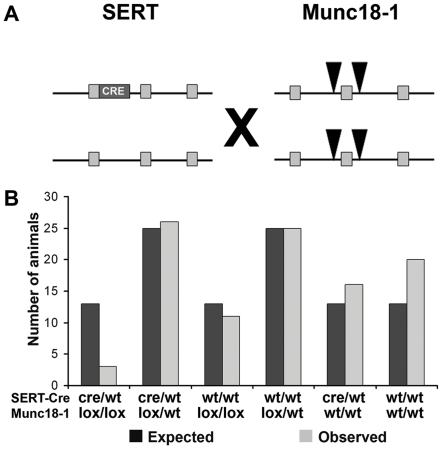
Postnatal lethality in SERT-Cre^cre/wt^ Munc18-1^lox/lox^ mice. (A) Genotype of the conditional knockout mice. Crossing SERT-Cre with Munc18-1^lox/lox^ mice results in deletion of Munc18-1 only and specifically in SERT expressing neurons, whereas all other neurons still express Munc18-1. (B) Genotyping mice after weaning at three weeks of age revealed that only few SERT-Cre^cre/wt^ Munc18-1^lox/lox^ mice survived up to three weeks.

To assess the effect of deletion of Munc18-1 in 5-HT neurons, we crossed Munc18-1^lox/lox^ mice with SERT-Cre mice, which express Cre in SERT expressing neurons. These are the 5-HT neurons in the raphe nuclei, but also some hippocampal neurons and thalamocortical neurons which express SERT transiently during development [15], [16]. Crossing SERT-Cre mice with Munc18-1^lox/lox^ mice results in mice in which Munc18-1 is specifically removed in SERT expressing neurons (Fig. 1A). We crossed SERT-Cre^cre/wt^ Munc18-1^lox/wt^ mice with SERT-Cre^wt/wt^ Munc18-1^lox/wt^ mice which should result in 12.5% of offspring which have SERT-Cre^cre/wt^ and Munc18-1^lox/lox^ genotypes. Genotyping 101 mice three weeks after birth revealed that genotype frequencies were not distributed at Mendelian ratio (p = 0.027). Only three SERT-Cre^cre/wt^ Munc18-1^lox/lox^ mice were found, whereas based on Mendelian ratio the expected number of SERT-Cre^cre/wt^ Munc18-1^lox/lox^ mice should be 13, giving a 77% mortality rate in SERT-Cre^cre/wt^ Munc18-1^lox/lox^ mice (Fig. 1B). These three remaining SERT-Cre^cre/wt^ Munc18-1^lox/lox^ mice all died in the fourth postnatal week. This shows that Munc18-1 deletion in SERT expressing neurons results in postnatal lethality.

### 5-HT neurons are initially generated but then quickly degenerate

**Figure 2:**
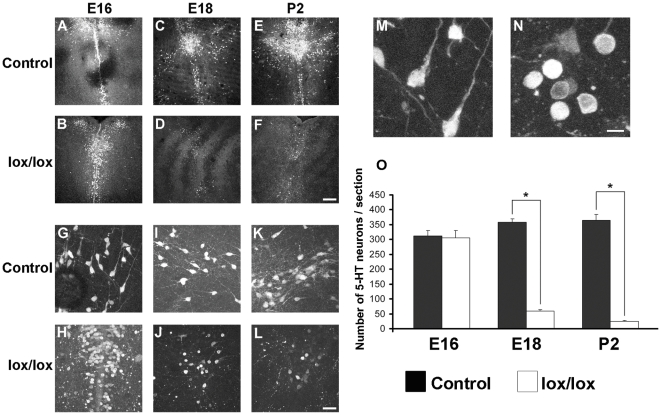
Rapid degeneration of 5-HT neurons in SERT-Cre^cre/wt^ Munc18-1^lox/lox^ mice. We compared the number of 5-HT cell bodies in the DRN in control and SERT-Cre^cre/wt^ Munc18-1^lox/lox^ mice at E16, E18 and P2. (A-F) At E16, in both control mice (A) and SERT-Cre^cre/wt^ Munc18-1^lox/lox^ (lox/lox) mice (B) the 5-HT cell bodies are distributed in the DRN topology. At E18 and P2, however, in midbrain sections from SERT-Cre^cre/wt^ Munc18-1^lox/lox^ brains (D, F respectively) only few 5-HT neurons are present compared to control sections (C, E respectively). (G-L) Zooming in on 5-HT cell bodies shows that in control sections at E16 (G), E18 (I) and P2 (K) the cell bodies grow out several neurites and have a fusiform or ovoid morphology. However, remaining 5-HT cell bodies in SERT-Cre^cre/wt^ Munc18-1^lox/lox^ sections at E16 (H), E18 (J) and P2 (L) have a round morphology with hardly any neurites. (M,N) Blow up of some 5-HT cell bodies in control and SERT-Cre^cre/wt^ Munc18-1^lox/lox^ mice sections shows the differences in 5-HT cell body morphology. (O) Analysis of the number of 5-HT cell bodies in the sections showed that there is no difference at E16, but at E18 and P2 there is a decrease of 80% to 90% in the number of 5-HT cell bodies in SERT-Cre^cre/wt^ Munc18-1^lox/lox^ sections. Scale bars: 200 µm in F, 50 µm in L and 20 µm in N. Data shown are mean ± standard error of the mean (SEM). * p<0.05.

To assess the effect of Munc18-1 deletion in 5-HT neurons on the development of the 5-HT system, we made coronal sections of paraformaldehyde (PFA) fixed brains at different developmental stadia and performed immunohistochemistry for 5-HT. As developmental time points we chose E16, E18 and postnatal day (P) 2. Expression of SERT, and thus of Cre recombinase, starts at E11 and the earliest recombination observed in the DRN in SERT-Cre mice was at E12.5 [16]. Since it will take some days before all remaining Munc18-1 mRNA and protein is degraded, we chose E16 as the first time point. We first focused on the 5-HT cell bodies in the DRN. Immunohistochemistry for 5-HT on brain slices from E16 SERT-Cre^cre/wt^ Munc18-1^lox/lox^ and control mice showed that in the DRN 5-HT neurons are present and these showed the characteristic DRN topology (Fig. 2A,B). The number of 5-HT neurons did not differ from control (control 312±17.67, SERT-Cre^cre/wt^ Munc18-1^lox/lox^ 305±25.43, Fig. 2O). However, morphological analysis of the neurons showed that these were already degenerating, as assessed by the round morphology and reduced number of primary neurites (Fig 2G,H). At E18, there was a ∼80% decrease in the number of 5-HT neurons in SERT-Cre^cre/wt^ M18^lox/lox^ mice compared to control (control 358±10.82, SERT-Cre^cre/wt^ Munc18-1^lox/lox^ 59±4.84, Fig. 2C,D,O). At P2 there were only few 5-HT neurons left in the DRN (control 364±19.68, SERT-Cre^cre/wt^ Munc18-1^lox/lox^ 25±2.89, Fig. 2E,F,O). At E18 and P2 the remaining 5-HT neurons in SERT-Cre^cre/wt^ Munc18-1^lox/lox^ brain sections displayed an altered morphology compared to control 5-HT neurons (Fig. 2I,J,K,L). 5-HT neurons in control brains had a characteristic fusiform or ovoid shape and grew out several primary neurites (Fig. 2M). In contrast, the mutant 5-HT neurons had a rounded morphology and the majority did not contain primary neurites, indicative of degenerating neurons (Fig. 2N). This showed that between E16 and P2 there is a massive degeneration of 5-HT neurons in the DRN.

### 5-HT projections innervate the DRN and cortex but are degenerated at P2

**Figure 3:**
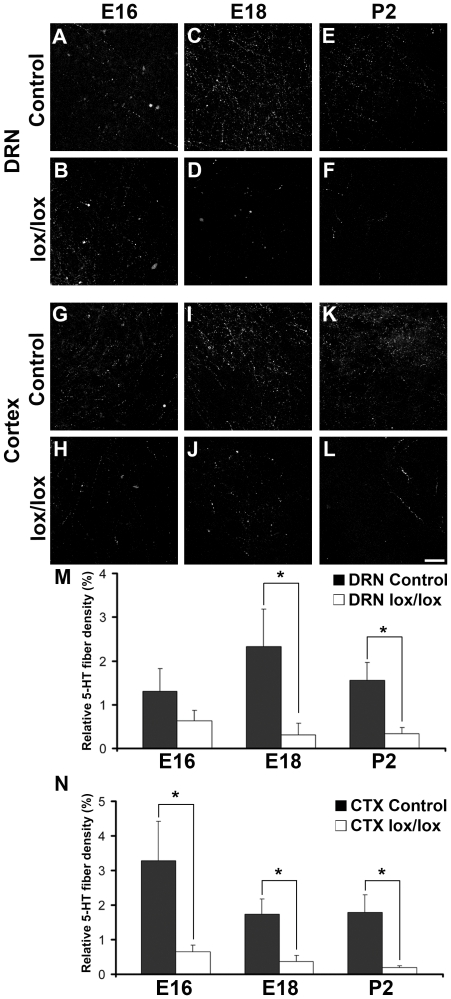
Degeneration of 5-HT projections in DRN and cortex. (A**–**F) At E16 in the midbrain containing the DRN, there was no difference in 5-HT projection density between control (A) and SERT-Cre^cre/wt^ Munc18-1^lox/lox^ (lox/lox) (B) mice. In control sections at E18 (C) and P2 (E) several 5-HT projections were present. However, in SERT-Cre^cre/wt^ Munc18-1^lox/lox^ sections at E18 (D) and P2 (F) only very few 5-HT projections are left. (G–L) In the cortex already at E16 there is a reduction in 5-HT projection density in sections from SERT-Cre^cre/wt^ Munc18-1^lox/lox^ (H) mice compared to control (G). At E18 (J) and P2 (L), in cortical sections from SERT-Cre^cre/wt^ Munc18-1^lox/lox^ mice only very few remaining 5-HT projections are present, in contrast to E18 (I) and P2 (K) control cortical sections. (M) Quantification of the 5-HT projection density in the DRN revealed that at E16 there is no difference, but at E18 and P2 5-HT projection density is reduced to ∼20%. (N) In cortical sections, at E16 and E18 the 5-HT projection density is reduced to ∼20% and at P2 the 5-HT projection density is reduced to ∼10%. Scale bar 50 µm in L. Data shown are mean±SEM * p<0.05.

Next, we focused on the innervation of the midbrain and cortex with 5-HT projections. Midbrain and cortex are the first regions which are densely innervated with 5-HT projections during development. We quantified 5-HT projection density first in DRN midbrain sections at E16, E18 and P2. At E16 the sections from both control and SERT-Cre^cre/wt^ Munc18-1^lox/lox^ mice contained several 5-HT projections and there was no significant difference in 5-HT innervation density (1.31±0.51% and 0.63±0.24% respectively, Fig. 3A,B). At E18, in control sections the 5-HT innervation density was slightly increased compared to E16 (Fig. 3C). However, in SERT-Cre^cre/wt^ Munc18-1^lox/lox^ sections the 5-HT innervation density was decreased compared to control (control 2.32±0.86%, SERT-Cre^cre/wt^ Munc18-1^lox/lox^ 0.31±0.27%, Fig. 3D).

At P2 only few 5-HT projections were left in SERT-Cre^cre/wt^ Munc18-1^lox/lox^ sections (control 1.6±0.41%, SERT-Cre^cre/wt^ Munc18-1^lox/lox^ 0.33±0.14%, Fig. 3E,F). Next, we focused on the 5-HT innervation density in the cortex. This revealed that already at E16 there was a reduction in 5-HT innervation density in SERT-Cre^cre/wt^ Munc18-1^lox/lox^ sections (control 3.27±1.14%, SERT-Cre^cre/wt^ Munc18-1^lox/lox^ 0.65±0.19%, Fig. 3G,H). At E18 and P2 the reduction in 5-HT innervation density was augmented, with only very few 5-HT projections left at P2 in the cortex (E18 control 1.73±0.45%, SERT-Cre^cre/wt^ Munc18-1^lox/lox^ 0.36±0.18%, P2 control 1.78±0.52%, SERT-Cre^cre/wt^ Munc18-1^lox/lox^ 0.19±0.04%, Fig. 3I,J,K,L). Quantification showed that in the midbrain sections the 5-HT innervation density was reduced to ∼20% at E18 and P2 (Fig. 3M). In the cortex, the 5-HT innervation density was reduced to ∼20% at E16 and E18 and ∼10% at P2 (Fig. 3N).

In summary, deletion of Munc18-1 in 5-HT neurons resulted in rapid degeneration of 5-HT cell bodies and the absence of 5-HT projections in the postnatal brain.

### Development of the 5-HT system is not affected in SERT-Cre^cre/wt^ Munc18-1^lox/wt^ mice

**Figure 4:**
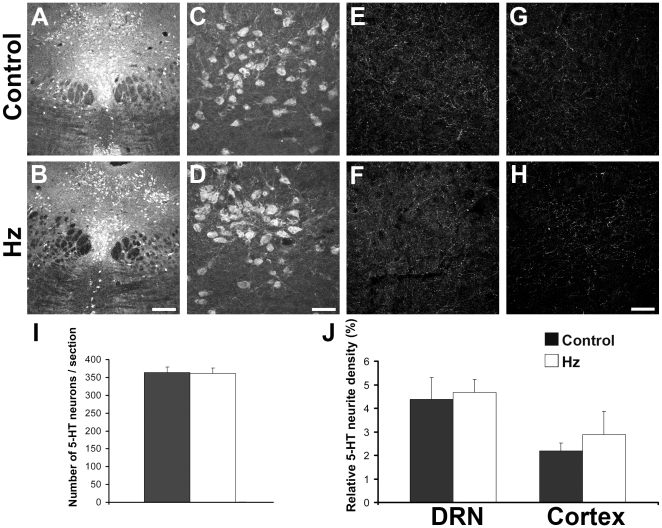
In SERT-Cre^cre/wt^ Munc18-1^lox/wt^ mice number of 5-HT cell bodies and 5-HT innervations are not affected. (A-D) Midbrain sections containing the DRN from control (A) and hz (B) mice shows that 5-HT neurons are distributed in the characteristic DRN topology. Zooming in on 5-HT cell bodies show that both in control (C) and hz sections (D) these neurons have a fusiform or ovoid morphology and grow out several neurites. (E–H) 5-HT projection density is not different between control and hz mice in the midbrain containing the DRN (E and F respectively) or in cortical sections from control and hz mice (G and H respectively). (I) Number of 5-HT cell bodies per section does not differ between control and hz. (J) 5-HT projection density in midbrain containing DRN and cortical sections is not different between control and hz. Scale bars: 200 µm in B, 50 µm in D and H.

Finally, we investigated whether deletion of one allele of Munc18-1 in SERT expressing neurons affected the number of 5-HT cell bodies in the DRN, or the 5-HT fiber density in the DRN and cortex. Analysis of midbrain sections from control and SERT-Cre^cre/wt^ Munc18-1^lox/wt^ (hz) mice showed that 5-HT neurons were distributed in the characteristic DRN topology, and control and hz were indistinguishable (Fig. 4A,B). 5-HT cell bodies in both control and hz had a fusiform or ovoid shape (Fig. 4C,D). Quantification of the number of 5-HT cell bodies in the DRN revealed no difference between control and hz (control 364±14.78, hz 361±15.01, Fig. 4I). Likewise, the 5-HT projection density in the midbrain and cortex were similar (midbrain control 4.38±0.93%, hz 4.67±0.55%; cortex control 2.18±0.35%, hz 2.87±0.99%, Fig. 4E–H,J). Thus, deletion of one allele of Munc18-1 does not affect 5-HT neuronal survival, 5-HT neurite outgrowth or 5-HT projection density.

## Discussion

In this study, we showed that SERT-Cre^cre/wt^ Munc18-1^lox/lox^ mice display a postnatal lethality phenotype, accompanied by a rapid degeneration of the 5-HT system. Inactivation of one allele of Munc18-1 in SERT expressing neurons does not affect 5-HT cell number or 5-HT innervation density.

Crossing Munc18-1^lox/lox^ mice with SERT-Cre mice results in deletion of Munc18-1 from SERT expressing cells. It was shown previously that crossing SERT-Cre mice with loxSTOPlox-EYFP mice results in >99% of 5-HT neurons in the DRN which are EYFP positively labelled [15]. However, SERT is also transiently expressed in some other brain regions during development, such as the thalamocortical neurons [16], [17]. In SERT-Cre ROSA-loxSTOPlox-LacZ double positive mice, strong LacZ expression was not only observed in the DRN, but also in the thalamus, cingulate cortex and in the CA3 region of the hippocampus [15], . Therefore, in SERT-Cre Munc18-1^lox/lox^ mice also in some non-5-HT neurons Munc18-1 will be deleted. Genotyping sacrificed mice at E16 or E18 revealed that there were more SERT-Cre^cre/wt^ Munc18-1^lox/lox^ mice than expected, showing that these mice do not die during embryonic development. However, genotyping mice three weeks after birth showed that the majority of SERT-Cre^cre/wt^ Munc18-1^lox/lox^ mice had died, and the remaining SERT-Cre^cre/wt^ Munc18-1^lox/lox^ mice died within the fourth postnatal week.

Previously we showed that in Munc18-1 KO mice the neuromuscular junction initially develops, but at later stages most of the motor neuronal cell bodies in the spinal cord degenerate [18]. In analogy with this, in the absence of regulated secretion the hypothalamo-neurohypophysial system is initially normally formed, but in later stages of development degenerates [19]. This is consistent with our observation that Munc18-1 is dispensable for initial generation and outgrowth of 5-HT neurons, but required for their survival.

Since also in some other neurons Munc18-1 is deleted, it is unclear whether the observed phenotypes can be attributed to the degeneration of the 5-HT system. In Tph2 KO mice, 5-HT neurons are completely devoid of 5-HT, yet there are no alterations in 5-HT neuron morphology and 5-HT neurite distribution [7]. Although these mice can survive into adulthood, approximately 50% does not survive the first four weeks and surviving mice have growth retardation [8]. Additionally, in a conditional Lmx1b KO mouse, 5-HT neurons are initially formed but almost all central 5-HT neurons fail to survive and brain 5-HT levels are reduced to ∼10% [5]. Remarkably, these mice display normal locomotor behaviour and show no abnormalities in gross brain morphology. Recently, it was shown that these mice have severe apnea and have a ∼20% mortality rate during the neonatal period, although the majority of these mice survive beyond P28 [20], [21].

Apparently, even the absence of central 5-HT synthesis or an almost complete removal of 5-HT neurons does not result in a severe (postnatal) lethality phenotype. Thus, although several of these mice display high (postnatal) mortality, the phenotypes observed are not as severe as in the SERT-Cre^cre/wt^ Munc18-1^lox/lox^ mice.

On the other hand, mice which lack GAP43 display disrupted barrel cortex formation and thalamocortical connections fail to form. These mice display a postnatal lethality phenotype, with 50% of homozygotes which die between P0 and P2, and >95% of homozygotes which die before P21 [22]. Although these mutant mice display a reduced 5-HT innervation in the cortex and hippocampus, they have no significant difference in the number of 5-HT neurons, and other areas such as the piriform cortex and amygdale receive normal 5-HT innervation [23]. Thus, based on these data it is unclear whether the postnatal lethality phenotype observed in this study is attributable to a degeneration of thalamocortical or 5-HT neurons. It would be very interesting to delete the Munc18-1 gene specifically in 5-HT neurons using the Pet-Cre transgenic line, in which expression of Cre is restricted to 5-HT neurons [24]. This approach could be used to investigate whether the phenotype we observed could indeed be attributed to degeneration of the 5-HT system rather than degeneration of thalamocortical neurons.

In conclusion, deletion of both alleles of Munc18-1 in SERT expressing neurons results in a rapid degeneration of the 5-HT system and postnatal lethality.

## Materials and Methods

### Laboratory animals

Generation and characterization of SERT-Cre mice has been described previously [15]. Briefly, immediately upstream of the SERT translational start codon a cassette containing Cre and a FRT-flanked neomycin cassette was inserted. To generate conditional Munc18-1 mice [14], floxed Munc18-1 mice were generated by insertion of LoxP sites flanking the second exon using homologous recombination. To obtain SERT-Cre^cre/wt^ Munc18-1^lox/lox^ mice, SERT-Cre^cre/wt^ Munc18-1^lox/wt^ mice were crossed with SERT-Cre^wt/wt^ Munc18-1^lox/wt^ mice. Pregnant females were sacrificed by cervical dislocation. Mouse embryos were obtained by caesarean section of pregnant females from timed matings. To investigate the effect of deletion of Munc18-1 in the 5-HT system on the development of the 5-HT system, SERT-Cre^cre/wt^ Munc18-1^lox/lox^ mice were used. SERT-Cre^wt/wt^ Munc18-1^lox/lox^, SERT-Cre^wt/wt^ Munc18-1^lox/wt^, or SERT-Cre^cre/wt^ Munc18-1^wt/wt^ littermates were not different from SERT-Cre^wt/wt^ Munc18-1^wt/wt^ mice and were used as controls. SERT-Cre mice were genotyped as has been previously described [15]. For genotyping of Munc18^lox/lox^ mice the following primers were used: 5′-ttggtggtcgaatgggcaggtag-3′, 5′-cctgtatgggtactgttcgttcactaaaata-3′ and 5′-ttctgaacttgaggccagtctgagacacag-3′. These studies were approved by the institutional ethical committee of the VU University (Protocol FGA 06-11-2). Animals were housed and bred according to institutional and Dutch guidelines.

### Immunohistochemistry

For immunohistochemical analysis of 5-HT, embryonic mouse brains were dissected and immediately fixed by immersion in 4% PFA in phosphate buffered saline (PBS, pH 7.4). For analysis of 5-HT neurons and projections in adult brains, mice were anaesthetized and transcardially perfused with 4% PFA in PBS. For post-fixation, brains were incubated in 4% PFA in PBS overnight. For cryoprotection, brains were incubated in increasing concentrations of sucrose. Subsequently, coronal slices of 40 µm were made. For immunohistochemistry, brain slices were first incubated in blocking buffer containing PBS supplemented with 0.5% Triton X-100 and 10% normal goat serum for two hours. Subsequently, slices were incubated overnight with primary antibodies in PBS containing 0.5% Triton X-100 at 4°C under gentle agitation. The next day, the slices were washed three times two hours in PBS and incubated with secondary antibodies in PBS for one hour under gentle agitation. Finally, slices were washed again three times two hours in PBS and mounted in Dabco Mowiol for analysis. All procedures were performed at room temperature unless otherwise stated. Polyclonal anti-5-HT (1:1000) (Immunostar/Diasorin) was used as the primary antibody, and as a secondary antibody GAR-546 (1∶1000) was used (Invitrogen). To analyze number of 5-HT cell bodies in DRN sections, at least three sections per experimental group were imaged using a CLSM 510 microscope. Number of 5-HT cell bodies was manually counted in the slices. For analysis of 5-HT fiber density in DRN and cortex Z-stacks of at least three positions per group was made. To analyze the 5-HT fiber density, the Z-stacks were projected in a single image and binarized. The 5-HT fiber density was quantified as the area in the image occupied by 5-HT projections compared to the total area.

### Statistical analysis

Data were analyzed using SPSS 17.0. For analysis of number of 5-HT cell bodies in DRN and 5-HT projection density in DRN and cortex, data was analyzed using the independent samples t-test. To analyze whether observed genotype frequencies differed significantly from a Mendelian ratio, a one-way chi-square test was performed. Data shown are mean±SEM. Significance level was set at p<0.05.
